# Supplementary material for: Inducing TRIB2-targeted protein degradation to reverse chemoresistance in acute myeloid leukaemia
Source: Biochem J. 2026 Mar 30;483(4):511–25. doi: 10.1042/BCJ20253463 (PMC13094653; doi:10.1042/BCJ20253463)
Supplement: Supplementary Figures S1-S4 [file BCJ-2025-3463_supp.pdf]

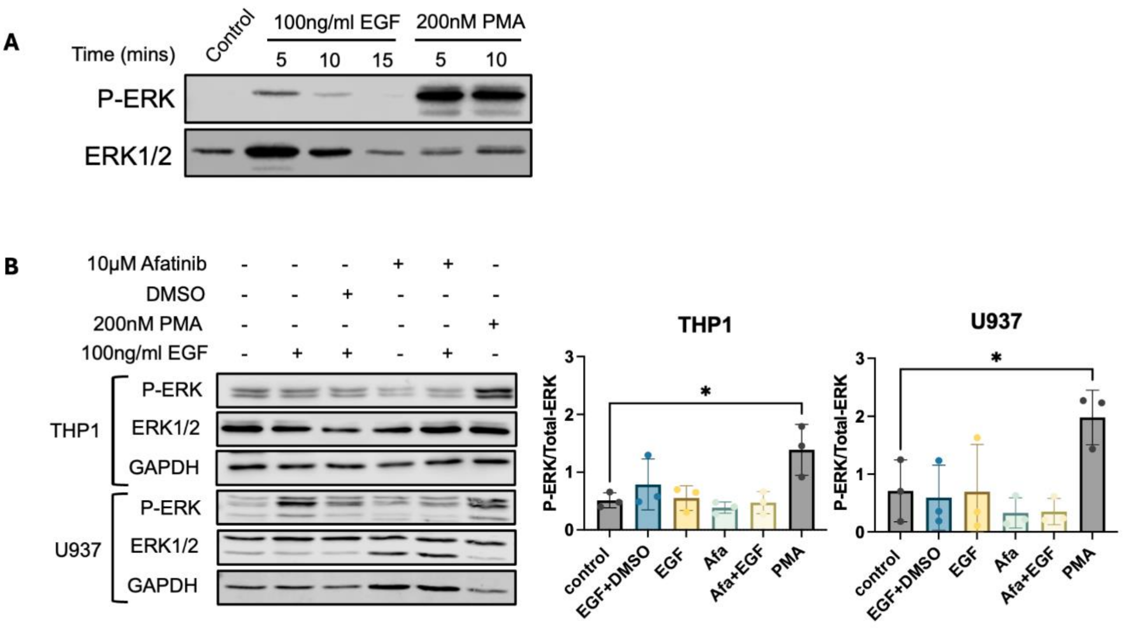
A
Time (mins)
Control
100ng/ml EGF
200nM PMA
5
10
15
5
10
P-ERK
ERK1/2
B
10µM Afatinib
DMSO
200nM PMA
100ng/ml EGF
THP1
P-ERK
ERK1/2
GAPDH
U937
P-ERK
ERK1/2
GAPDH
THP1
U937
P-ERK/Total-ERK
*
control
EGF+DMSO
EGF
Afa
Afa+EGF
PMA

Supplementary Figure 1
A) Western blot of EGF and PMA stimulated U937 cells showing phospho-ERK relative to total-ERK protein levels.
B) Representative western blots and averaged densitometry of EGF, Afatinib and PMA-stimulated U937 and THP1 cells showing phospho-ERK relative to total-ERK protein levels.

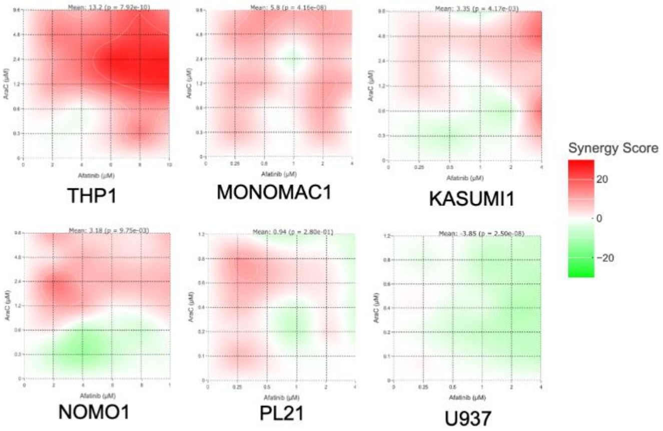
Mean: 13.2 (p = 7.92e-10)
AraC (µM)
Afatinib (µM)
THP1
Mean: 5.8 (p = 4.16e-08)
AraC (µM)
Afatinib (µM)
MONOMAC1
Mean: 3.35 (p = 4.17e-03)
AraC (µM)
Afatinib (µM)
KASUMI1
Mean: 3.18 (p = 9.75e-03)
AraC (µM)
Afatinib (µM)
NOMO1
Mean: 0.94 (p = 2.80e-01)
AraC (µM)
Afatinib (µM)
PL21
Mean: -3.85 (p = 2.50e-08)
AraC (µM)
Afatinib (µM)
U937
Synergy Score
20
0
−20

Supplementary Figure 2

Synergy maps establishing drug synergy between AraC and Afatinib at variable doses in AML cell lines.

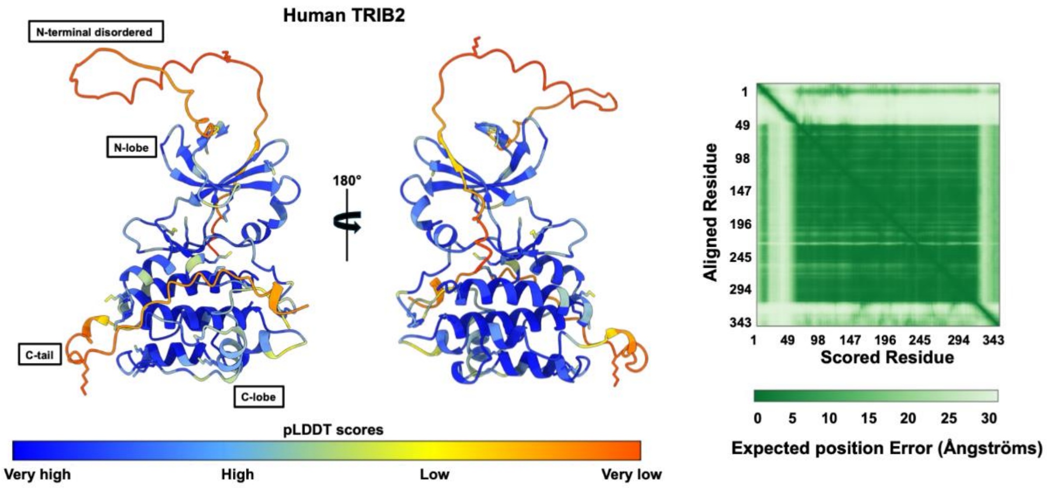
Human TRIB2
N-terminal disordered
N-lobe
180°
C-tail
C-lobe
pLDDT scores
Very high
High
Low
Very low
1
49
98
147
196
245
294
343
Aligned Residue
Scored Residue
0
5
10
15
20
25
30
Expected position Error (Ångströms)

Supplementary Figure 3

The top-ranked structure of full-length human TRIB2 [1-343] generated using AlphaFold 3 (pTM = 0.77) and visualized in UCSF ChimeraX, coloured by predicted local distance difference test (pLDDT) confidence score. Predicted alignment error (PAE) is shown at right.

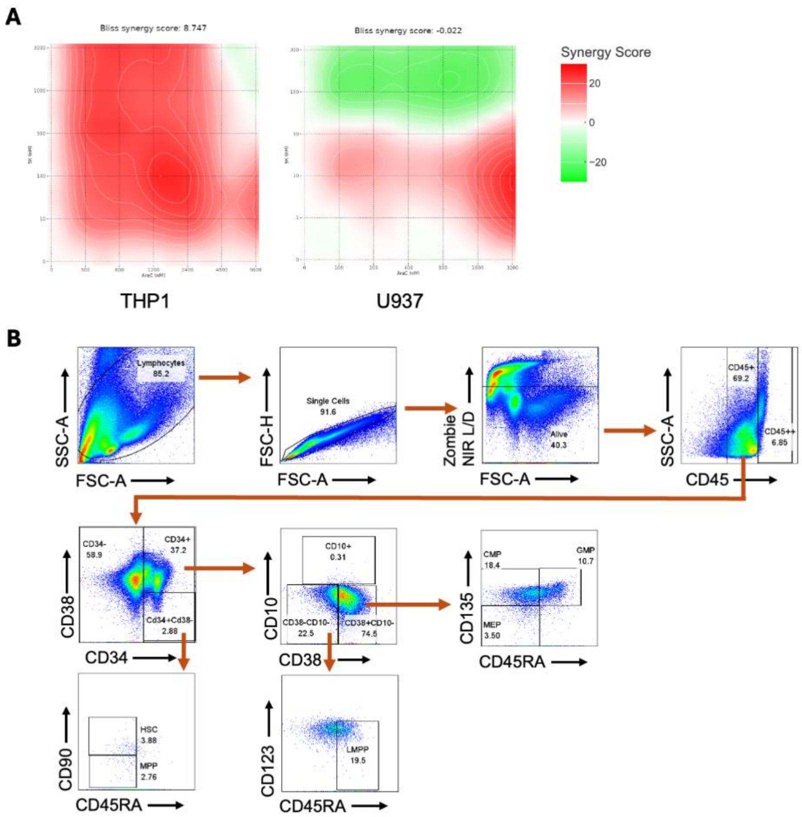
A
Bliss synergy score: 8.747
Bliss synergy score: -0.022
Synergy Score
20
0
-20
THP1
U937
B
Lymphocytes
85.2
SSC-A
FSC-A
Single Cells
91.6
FSC-H
FSC-A
Zombie NIR L/D
Alive
40.3
FSC-A
CD45+
69.2
CD45++
6.85
SSC-A
CD45
CD34-
58.9
CD34+
37.2
Cd34+Cd38-
2.88
CD38
CD34
CD10+
0.31
CD38-CD10-
22.5
CD38+CD10-
74.5
CD10
CD38
CMP
18.4
GMP
10.7
MEP
3.50
CD135
CD45RA
HSC
3.88
MPP
2.76
CD90
CD45RA
LMPP
19.5
CD123
CD45RA

Supplementary Figure 4

A) Synergy maps establishing drug synergy between AraC and 5K PROTAC at variable doses in THP1 and U937 cell lines.

B) Representative flow cytometry plots and gating strategy for analysis of hematopoietic stem and progenitor cell populations in 5K treated AML and cord blood samples. Antibody panel: CD34 (BD:562577), CD38 (BD:564498), CD45 (BD:3340905), CD45RA (BD:741010), CD90 (BioLegend:328124), CD10 (BD:555376), CD135 (BD:756417), CD123 (BD:567287), Zombie NIR fixable viability kit (BioLegend:423106)
